# Decoding Cerebrospinal Fluid: Integrative Metabolomics Across Multiple Platforms

**DOI:** 10.3390/mps9010008

**Published:** 2026-01-08

**Authors:** Antoine Presset, Sylvie Bodard, Antoine Lefèvre, Edward Oujagir, Camille Dupuy, Jean-Michel Escoffre, Lydie Nadal-Desbarats

**Affiliations:** 1 Imaging Brain & Neuropsychiatry iBrain U1253, Université de Tours, INSERM, 37032 Tours, France; sylvie.bodard@inserm.fr (S.B.); antoine.lefevre@univ-tours.fr (A.L.); edward.oujagir@univ-tours.fr (E.O.); camille.dupuy@univ-tours.fr (C.D.); 2 INSERM, Neurocentre Magendie U1215, 33077 Bordeaux Cedex, France; 3 Plateforme de Métabolomique et d’Analyses Chimiques, US61 ASB, Université de Tours, CHRU Tours, INSERM, 37032 Tours, France; 4 Centre d’Étude des Pathologies Respiratoires CEPR U1100, Université de Tours, INSERM, 37032 Tours, France

**Keywords:** cerebrospinal fluid (CSF), metabolomics, ^1^H-NMR spectroscopy, HPLC-MS, biomarkers, biochemical pathways

## Abstract

Cerebrospinal fluid (CSF) is a key biological matrix that reflects the physiological and pathological states of the central nervous system (CNS). It supports brain function by regulating ionic balance, facilitating molecular transport, and clearing metabolic waste. In this article, we present a standardized protocol for CSF collection along with an integrative multiplatform metabolomic workflow that combines proton nuclear magnetic resonance spectroscopy (^1^H-NMRS) and high-performance liquid chromatography coupled to mass spectrometry (HPLC-MS). Integrating these complementary analytical modalities enhances metabolite coverage and improves analytical robustness, enabling a more comprehensive and reliable characterization of the CSF metabolome. This workflow supports the discovery of potential biomarkers and advances our understanding of neurochemical alterations within the CNS.

## 1. Introduction

The meninges are three protective membranes surrounding the brain and spinal cord: the dura mater, arachnoid membrane, and pia mater [1,2]. Cerebrospinal fluid (CSF) is a clear, colorless liquid located within the ventricles and subarachnoid spaces. In adult humans, the total CSF volume is approximately 150 mL (about 25 mL within the ventricles and 125 mL in the subarachnoid spaces). CSF is primarily secreted by the choroid plexuses in the ventricles, although the brain interstitial fluid, the ependyma, and cerebral capillaries also contribute to its composition [3]. CSF circulation is mainly driven by the arterial pulse wave, but respiration, posture, venous pressure, and physical activity also influence its flow and pressure dynamics [4]. CSF provides hydromechanical protection, regulates interstitial fluid composition, modulates neuronal activity, and plays a central role in maintaining cerebral homeostasis by clearing metabolic waste and regulating ionic balance [5]. Alterations in CSF dynamics or composition can therefore disrupt brain function [6]. The choroid plexus, located in the lateral, third, and fourth ventricles, is the principal site of CSF secretion. This vascularized structure is composed of choroidal epithelial cells, ependymal cells, endothelial cells, connective stroma, and a rich capillary network [7]. Choroidal epithelial cells, characterized by apical microvilli and tight junctions, actively transport ions and water to produce CSF. Ependymal cells, lining the ventricles and continuous with choroidal epithelium, contribute to regulating CSF volume and composition. Endothelial cells mediate exchanges between blood and CSF compartments [3,7]. CSF secretion involves (1) passive filtration of plasma into the choroidal interstitial space; (2) active ion transport across the choroidal epithelium via cytoplasmic carbonic anhydrase and ion carriers; (3) intracellular conversion of CO_2_ and H_2_O into H^+^ and HCO_3_^−^ by carbonic anhydrase; and (4) exchange of these ions for Na^+^ and Cl^−^ at the basolateral membrane. ATP-dependent pumps on the apical membrane subsequently transport Na^+^, Cl^−^, HCO_3_^−^, and K^+^ into the ventricular lumen, followed by osmotic water movement through aquaporin I channels [8]. Disturbances in CSF production, circulation, or absorption can lead to significant CSF dysfunctions, including hydrocephalus, intracranial pressure dysregulation, or infection [9]. As a result, CSF analysis is a critical tool for evaluating CNS disorders, enabling the detection of metabolic abnormalities and the identification of potential biomarkers.

Metabolomics—the systematic study of small molecules (metabolites) within a biological system—aims to identify and quantify these compounds to capture the organism’s functional state. Unlike genomics or proteomics, metabolomics provides a direct snapshot of the metabolic phenotype, reflecting the combined effects of the genetic, environmental, and biochemical factors [10]. This field primarily relies on mass spectrometry (MS) and nuclear magnetic resonance (NMR) spectroscopy, used either in targeted (specific metabolites) or untargeted (global profile) approaches depending on study objectives [11]. To the best of our knowledge, only a limited number of studies have employed an integrative NMR- and Liquid-Chromatography MS-based metabolomics approach in rats, regardless of the biological matrices analyzed, even though such integrative strategies leverage the complementary strengths of each technique. NMR enables the direct and non-invasive identification of metabolites, offering a substantial advantage for both qualitative and quantitative analysis. However, its sensitivity is often insufficient for detecting low-abundance metabolites. In contrast, Liquid Chromatography coupled to mass spectrometry (LC-MS) provides high sensitivity and can analyze a broad spectrum of metabolites with remarkable precision, although it typically requires more complex sample preparation and does not always offer the same level of direct structural identification as NMR. Recently Kim et al. (2023) reported an ex vivo NMR-based metabolomics approach using cerebrospinal fluid for the diagnosis of primary CNS lymphoma, correlating metabolic signatures with MR imaging features. Their findings suggest that while NMR metabolomics may aid in diagnosing primary central nervous system lymphoma (PCNSL), it is less informative for the prognosis [12]. To further strengthen such metabolomics investigations, one could envision that combining NMR and LC-MS would provide a more comprehensive characterization of metabolic alterations. Similarly, Locasale et al. (2012) studied changes in CSF constituents using high-throughput LC-MS/MS analysis in a small cohort of patients with malignant glioma compared with control subjects [13]. Using targeted MS-based metabolomics and unsupervised statistical techniques, they identified metabolic signatures, mainly involving polar metabolites (tryptophan’s derivative and citric acid cycle components), that distinguished glioma patients from controls. Additionally, Nielsen et al. (2021) characterized Alzheimer’s disease (AD) using an integrative NMR- and LC-MS-based metabolomics workflow. By combining these two methodologies, they identified multiple altered pathways related to AD pathology [14]. Current challenges in this field notably include the complexity of integrating datasets generated from these fundamentally different analytical platforms. Here, we describe a complete protocol for CSF collection in rats before and after a transient blood–brain barrier opening (BBBO) induced by microbubble-assisted ultrasound, coupled with a comprehensive multiplatform metabolomics workflow integrating ^1^H-NMR spectroscopy and HPLC-MS.

## 2. Experimental Design

**Figure 1:**
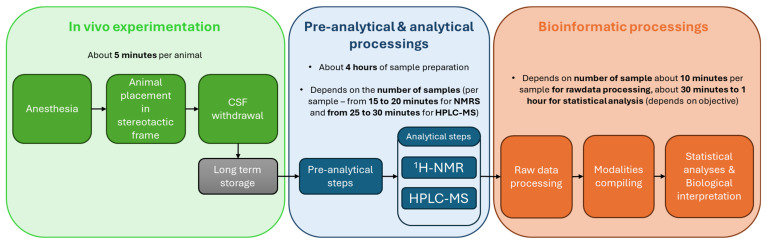
Schematic overview of the comprehensive multiplatform metabolomics workflow integrating ^1^H-NMR spectroscopy and HPLC-MS.

CSF is collected from anesthetized animals positioned in a stereotaxic frame using only ear bars. This method is adapted from Ceaglio et al. and Pegg et al. [15,16]. A maximum of 200 µL of CSF can be collected per rat. Samples are immediately placed on ice and centrifuged, and the supernatant is transferred to sterile Eppendorf tubes for storage at −80 °C. For downstream analyses, each CSF sample is aliquoted into three tubes corresponding to the different analytical modalities by the two platforms. Metabolite extraction procedures are adapted to each modality (i.e., ^1^H-NMR and HPLC-MS using a C18 column or HPLC-MS using an HILIC column), including a protein precipitation step using methanol (MeOH). During this pre-analytical step, quality control (QC) samples—either pooled study samples or reference materials—are used in metabolomics workflows to monitor and evaluate the variability and reliability of analytical platforms throughout the entire experiment. QC samples ensure data consistency, enable the detection of technical variability, and help identify potential systematic errors during sample acquisition. To assess analytical performance, several QCs should be injected at the beginning and the end of each analytical run, as well as after every ten samples (depending on the total sample set). Preparing QCs requires calculating the appropriate pooling volume from each individual sample and processing the pooled aliquots in the same manner as all study samples. Our protocol primarily targets polar metabolites to generate a rapid and representative snapshot of the metabolome (e.g., amino acids, carbohydrates, tricarboxylic acids, etc.). Metabolite identification and quantification are achieved using the ASICS R package (R version 4.5.2—ASICS package version 2.24.0) for NMR spectra and the XCalibur software (version 2.2) for HPLC-MS data. Results are exported as tables of relative metabolite intensities or abundancies. Finally, statistical analyses—multivariate, univariate, and pathway integration—are conducted to interpret metabolomic profiles (Figure 1).

### 2.1. Materials

1. Kinetex XB C18 (Phenomenex Incorporation; Torrance, CA, USA).
2. Cortecs HILIC (Waters Corporation; Milford, MA, USA).
3. Mass Spectroscopy Metabolite Library of Standards (IROA Technologies, Sea Girt, NJ, USA).
4. 25G sterile winged infusion set (SV*25NL30; Terumo, Tokyo, Japan).
5. 1 mL sterile syringe (MDSS01SE; Terumo, Tokyo, Japan).
6. 500 µL sterile Eppendorf tubes.
7. Ethanol (EtOH) 70% (*v*/*v*).
8. Betadine.
9. Electric shaver.
10. Stereotaxic frame.
11. Anesthetic (ketamine/xylazine or isoflurane).
12. Acetonitrile, HPLC-MS grade.
13. Methanol (MeOH), HPLC-MS grade.
14. MilliQ water (18.2 MΩ.cm at 25 °C, TOC 3 ppb, <1 particle/mL).
15. Trimethylsilyl propionate (TSP).

### 2.2. Equipment

- Stereotaxic frame model 963 (Kopf Instruments; Tujunga, CA, USA).
- Ultra High-Performance Liquid Chromatography (UPLC) Ultimate WPS-3000 (Dionex; Sunnyvale, CA, USA).
- Q-Exactive hybrid quadrupole-Orbitrap mass spectrometer (Thermo Fisher Scientific; Bremen, Germany).
- DRX-600 Avance III HD NMR spectrometer with Triple Resonance Inverse Cryoprobe (Bruker; Billerica, MA, USA).

## 3. Procedure

### 3.1. CSF Withdrawal

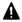

CRITICAL STEP At least two experimenters are required—one to operate the syringe and the other to stabilize the winged needle.
  1. Anesthetize the animal using a ketamine/xylazine mixture (70 mg/kg and 7 mg/kg, respectively). Alternative approved anesthetic methods may also be used.
  2. Place it in the stereotaxic frame with only ear bars.
  3. Tilt the head downward at a 15° to 20° angle relative to the vertical axis to expose the foramen magnum at the C1 vertebra (atlas).
  4. Secure the head with the tooth bar.
  5. Shave, depilate, and clean the neck with 70% ethanol followed by betadine.
  6. Identify the puncture site at the intersection of two lines: one connecting the external occipital protuberance to the spinal column and the other joining the two mastoid processes.
  7. Apply gentle negative pressure by slightly pulling the syringe plunger during insertion.
  8. Position the winged needle at a 90° angle relative to the horizontal plane of the skin surface at the sampling site. Ensure that the orientation of the wings is parallel to the ear bars.
  9. Collect a maximum of 200 µL of CSF. This is the maximum volume recommended prior to sacrifice to avoid physiological disturbance.

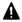

CRITICAL STEP If any red blood cells (RBCs) enter the winged needle tubing, the second experimenter must immediately clamp and then cut the tube. Cutting the tube without prior clamping may generate suction, which could draw additional RBCs into the system.
  10. Immediately place the collected CSF on ice.
  11. Centrifuge the sample at 10,000× *g* for 15 min at 4 °C to remove any cellular debris.
  12. Transfer the supernatant to a clean, sterile Eppendorf tube.
  13. Store the processed CSF samples at −80 °C until further analysis.

### 3.2. Pre-Analytical Procedure

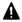

CRITICAL STEP Aliquot each sample into two 50 µL tubes and one 100 µL tube for the three different analytical modalities. Include quality control (QC) samples to assess analytical platform variation: several QC samples should be analyzed at the beginning and end of each run and after every 10 samples.

#### 3.2.1. HPLC-MS

1. Pipette 50 µL of each sample into a clean microcentrifuge tube.
2. Add 300 µL of cold methanol (–20 °C).
3. Vortex for 5 s to ensure proper mixing.
4. Incubate at −20 °C for 30 min to precipitate proteins.
5. Centrifuge at 5000× *g* for 25 min at 4 °C.
6. Carefully collect a fixed volume of supernatant (e.g., 250 µL), ensuring that the pellet remains undisturbed.
7. Transfer to new tubes and evaporate to dryness using a vacuum concentrator (35 °C, 3 h).
8. Reconstitute dried extracts for the following:
  a. HILIC column: 100 µL of acetonitrile/water (9:1, *v*/*v*).
  b. C18 column: 100 µL of methanol/water (1:9, *v*/*v*).
9. Vortex briefly to dissolve completely.
10. Transfer reconstituted samples to HPLC vials for injection and analysis.

#### 3.2.2. ^1^H-NMR

1. Dilute the CSF sample 1:4 by cold methanol (–20 °C) to a final volume of 200 µL.
2. Vortex briefly.
3. Centrifuge at 4000× *g* for 10 min at 4 °C to pellet precipitated proteins.
4. Transfer a fixed volume of supernatant (e.g., 80 µL) into a clean hemolysis tube.
5. Dry under vacuum using a SpeedVac (35 °C, 2 h).
6. Immediately before acquisition, reconstitute the dried extract in 200 µL of phosphate buffer (prepared in D_2_O) with 10 µL of 3.2 mM of TSP as a chemical shift reference (leading to a 152 μM final concentration in the NMR tube).
7. Vortex gently to ensure full dissolution.
8. Transfer to NMR tube and proceed with data acquisition.

### 3.3. Analytical Procedure

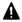

CRITICAL STEP Preparation steps may vary depending on the equipment used. All samples must be analyzed simultaneously on the same analytical platform to ensure comparability. Three aliquots were prepared: the first for C18 HPLC-MS, the second for HILIC HPLC-MS, and the third for NMR analysis. For HPLC-MS experiments, it is essential to first construct a commercial reference library as described in the Reference Library Construction Section.

#### 3.3.1. HPLC-MS

##### Chromatographic Column Setup

*C18 Column Conditions*

1. Set up the UPLC Ultimate WPS-3000 system equipped with a Kinetex XB C18 (150 mm × 2.1 mm, 1.7 µm) under the following conditions:
  - Column temperature: 55 °C.
  - Mobile phase A: Milli-Q water with 0.1% (*v*/*v*) formic acid.
  - Mobile phase B: Methanol with 0.1% (*v*/*v*) formic acid.
  - Flow rate: 0.2 mL/min.
  - Gradient duration: 28 min for both positive and negative ionization modes.
2. Run analyses by injecting samples into a mass spectrometer configured as described in the Mass Spectrometer Analysis Section.

*HILIC Column Conditions:*

1. Prepare a UPLC Ultimate WPS-3000 system equipped with a Cortecs HILIC (150 mm × 2.1 mm, 1.6 µm) under the following conditions:
  - Column temperature: 55 °C.
  - Mobile phase A: Milli-Q water with 10 mM of ammonium formate.
  - Mobile phase B: Acetonitrile with 10 mM of ammonium formate.
  - Flow rate: 0.2 mL/min.
  - Gradient duration: 22 min for positive ionization mode.
2. Run analyses by injecting samples into a mass spectrometer configured as described below.

##### Mass Spectrometer Analysis

1. Configure the Q-Exactive hybrid quadrupole-Orbitrap mass spectrometer for both positive and negative electrospray ionization (ESI) mode as follows:
  - Spray voltage: ±3000 V.
  - Capillary temperature: 325 °C.
  - Heater temperature: 350 °C.
  - Sheath gas flow: 25 arbitrary units (AU).
  - Auxiliary gas flow: 8 AU.
  - Sweep gas flow: 3 AU.
  - S-Lens RF level: ±100 V.
2. Configure the analyzer and the detector as follows:
  - Acquisition mode: Full scan.
  - Mass range: 58–870 m/z.
  - Resolution: 70,000 at m/z 200.
  - AGC target: 1 × 10^6^ charges.
  - Maximum injection time: 250 ms.

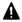

CRITICAL STEP QC samples must be injected at the beginning and end of the run and after every 10 samples to monitor analytical stability.

3. Inject samples in the analytical platforms.
  *a.*  Injection volume:
    - A total of 5 µL for C18 column.
    - A total of 10 µL for HILIC column.

#### 3.3.2. ^1^H-NMR

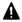

CRITICAL STEP As for HPLC-MS, QC samples should be analyzed first and last in the sequence, and after every 10 samples, to ensure data quality and reproducibility.
  1. Place each NMR tube in a rack and insert the rack into the sample changer of the Bruker DRX-600 Avance III HD spectrometer (Bruker, Billerica, MA, USA).
  2. The probe must be tuned to the proper frequency to detect the signal of ^1^H.
  3. Lock the magnetic field on the deuterium resonance from the solvent used (here, D_2_O + H_2_O), followed by shimming to optimize field homogeneity.
  4. Set up the acquisition parameters: pulse sequence with water suppression, 90° pulse, sweep width, relaxation delay, number of dummy scans, number of scans, and time domain.
  5. Start the NMR acquisition.

### 3.4. Data Processing

#### 3.4.1. HPLC-MS

##### Reference Library Construction

1. Use the Mass Spectroscopy Metabolite Library of Standards (IROA Technologies, Sea Girt, NJ, USA), containing 610 standard metabolites, to build an in-house spectral reference library.
2. Analyze these standards under the same chromatographical and mass spectrometry conditions as the biological samples (as described in Section 3.3.1).

##### Metabolite Identification

1. Identify metabolites in biological samples by matching spectral features against the in-house reference library.
2. Verify by using the XCalibur 2.2 software (Thermo Fisher Scientific, San Jose, CA, USA) for metabolite identification according to the following four criteria:
  - Retention time must be within ±20 s of the standard.
  - Measured mass must be within ±10 ppm of the theoretical mass.
  - Peak shape and isotopic distribution must be consistent with the reference spectrum of the standard.
  - Metabolites exhibiting a relative standard deviation greater than 30% are excluded from the final dataset. Metabolites with a signal-to-noise ratio (SNR) < 3 are discarded.

##### Peak Integration and Intensity Extraction

1. For metabolites meeting all four identification criteria, integrate the area under the corresponding chromatographic peak to determine intensity.
2. Export results as a data matrix containing metabolite identifiers and their intensities across all samples.

#### 3.4.2. ^1^H-NMR

##### Free Induction Decay (FID) Processing

1. The obtained FID signal is converted into a spectrum by applying a Fourier transformation using TopSpin 3.6.2 (Bruker, Billerica, MA, USA).
2. Apply zero filling and exponential multiplication with a line broadening of 0.3 Hz.
3. Apply zero- and first-order phase correction.
4. Perform baseline flattening and align spectral peaks if necessary.
5. Normalize the spectra using the internal reference compound (TSP or another molecule).

##### Metabolite Identification and Quantification

1. Identify and quantify spectral peaks by deconvoluting and reconstructing NMR spectra using a library of pure compounds implemented in the ASICS R package [17,18].
2. Run the ASICS workflow to quantify a maximum of 190 metabolites.
3. Export results as a data table containing relative quantifications across all samples.

#### 3.4.3. Quality Control and Database Attributes

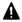

CRITICAL STEP All data matrices must be organized with metabolites as rows and samples as columns. QC samples must be analyzed concurrently with study samples.

1. Calculate the coefficient of variation (CV) of each metabolite within QC samples for all analytical platforms (C18 ESI+, C18 ESI-, HILIC+, and NMR) as follows:
  (1) $$CV\left(\%\right)=\frac{Standard\;Deviation\;in\;QC\;sample}{Mean\;in\;QC\;sample}\times 100$$
2. Remove all metabolites showing a CV greater than 30%.
3. Using metabolite databases (in-house database, KEGG, Reactome, HMDB, etc.), associate each metabolite to a unique identifier (e.g., glucose → KEGG ID: C00031).
4. Save curated data matrices for redundancy filtering.

### 3.5. Statistical Analysis

#### 3.5.1. Redundancy Filtering and Normalization

1. Compile a unified data table containing metabolites from each analytical modality and tag them with their corresponding modality (C18 ESI+, C18 ESI-, HILIC+, and NMR).
2. For metabolites detected by multiple platforms, retain the measure from the modality showing the lowest CV in the QC samples.
3. Remove QC samples from the data matrix and normalize CSF samples to the total ion or spectral area [19,20,21].

#### 3.5.2. Multivariate Analyses

1. Perform multivariate analyses using R and the ropls package (version 1.40.0) [22] or equivalent statistical software.
  a. Unsupervised analysis: perform a Principal Component Analysis (PCA) to explore clustering and detect potential outliers.
  b. Supervised analysis: perform a Partial Least Squares Discriminant Analysis (PLS-DA) to identify group discriminants.
2. Assess model performance using Q^2^, where values closer to 1 indicate stronger predictive ability.
3. Validate model significance through permutation testing (implemented in ropls), ensuring pR^2^Y and pQ^2^ are <0.05 and Q^2^ is >0.5.
4. Identify significant metabolites based on Variable Importance in Projection (VIP) scores. Select those with a VIP > 1 for further analyses.

OPTIONAL STEP Receiver Operating Characteristic (ROC) curves may be plotted to evaluate the predictive performance of discriminant metabolites, either individually or in combination [23]. The area under the curve (AUC) indicates the ability to correctly classify new samples.

#### 3.5.3. Univariate Analyses

1. Assess data normality using the Shapiro–Wilk test and homogeneity of variances using the Fligner–Killeen test.
2. Choose the appropriate statistical test (parametric or non-parametric) depending on these results to identify metabolites differing significantly between groups.
3. Adjust *p*-values for multiple testing using the Bonferroni correction [24] or other suitable methods.
4. Metabolites are considered significantly altered when the adjusted *p*-value is < 0.05.
5. Calculate the log_2_ Fold Change (Log2 FC) for all significantly different metabolites.

OPTIONAL STEP A volcano plot can be used to visualize metabolites according to their log_2_ fold change and statistical significance (−log_10_ *p*-value). Metabolites with a Log2 FC > 1 and a −log_10_ *p*-value > 1.30 were selected as candidates for further investigation.

#### 3.5.4. Biological Integration

1. Identify metabolites showing significant differences between experimental groups or use metabolites with a VIP > 1 and a |Log2 FC| > 1.
2. Determine affected metabolic pathways using KEGG identifiers and perform Over-Representation Analysis (ORA) via a hypergeometric test, which evaluates whether certain metabolites are statistically over-represented in a pathway.
3. A pathway is considered significantly impacted when the adjusted *p* is <0.05.
4. Manually inspect significantly dysregulated pathways and remove non-relevant ones (i.e., pathways containing metabolites without reaction biochemical relationships).

## 4. Expected Results

This section describes the raw and processed data obtained from a multiplatform CSF metabolomics study. A metabolomics workflow generates large and complex datasets: a three-dimensional dataset for HPLC-MS (intensity, retention time, and mass-to-charge ratio) and a two-dimensional dataset (intensity and chemical shift) for NMR spectroscopy. After data processing, metabolite identification and quantification yield structured tables. Because each analytical platform offers complementary yet sometimes overlapping information, redundancy filtering is required. Some metabolites are detectable on only one platform, while others are shared across several. Given the high dimensionality of the data (many metabolites, samples, and experimental conditions), multivariate statistical approaches are preferred. Unsupervised methods (e.g., PCA) enable exploration of inherent data structure, while supervised methods (e.g., PLS-DA or OPLS-DA) identify discriminant metabolites associated with experimental groups. Healthy Sprague–Dawley rats (Janvier Labs, Le Genest-Saint-Isle, France) were housed in an animal facility under a 12 h/12 h light/dark cycle with ad libitum access to food and water. Upon arrival, the rats were 7 weeks old. After a mandatory one-week acclimation period, their average body weight was 340 g (±12 g). Animals were randomly assigned to groups of four per cage. This workflow was applied to eight 2-month-old rats subjected to transient BBBO using microbubble-assisted ultrasound: four control animals and four animals sampled 3 h post-BBBO. This procedure consisted of transiently opening the BBB through the combined action of an intravenous injection of microbubbles and their activation by ultrasound generated by a probe positioned above the skull. Animals were placed in a stereotaxic frame, the skull was exposed, and bregma was used as a reference point to position the ultrasound transducer over the right striatum (anteroposterior: −0.5 mm; laterality: −3.15 mm; depth: 5 mm). A volume of 100 µL of microbubbles was injected via the caudal vein, followed by 100 µL of saline to flush the line. The target region was then exposed to 1 MHz ultrasound for 30 s using a pulse repetition frequency (PRF) of 1 Hz and 10,000 cycles per pulse, corresponding to a burst length (BL) of 10 ms, to transiently open the BBB. CSF was subsequently collected from anesthetized animals positioned in a stereotaxic frame using only ear bars. As this procedure was terminal, animals were euthanized after CSF sampling by administering a lethal dose corresponding to three times the anesthetic induction dose. All CSF withdrawals were successful, and no animal died during the collection procedure.

**Figure 2:**
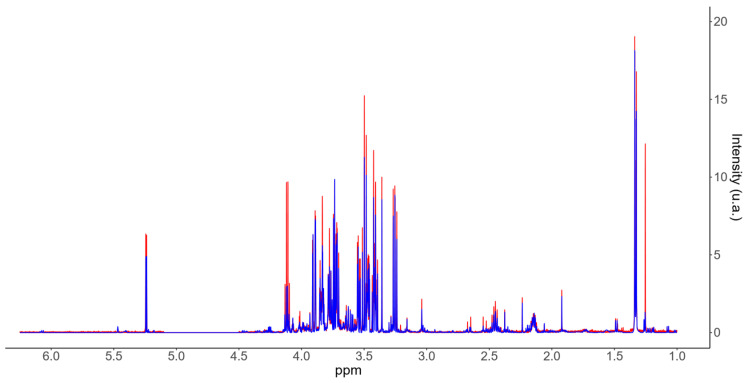
Representative ^1^H-NMR spectrum in CSF QC sample: original spectrum (red) and reconstructed spectrum (blue) after processing with the ASICS R package.

Raw NMR spectra undergo Fourier transformation followed by post-processing steps—phase and baseline correction—to enhance spectral quality and quantification accuracy (Figure 2). After processing, spectral peaks are identified and quantified using the ASICS R package, which reconstructs spectra based on a reference library of pure compounds [17,18]. This workflow quantifies up to 190 metabolites, producing a data table of relative metabolite intensities (or concentrations if normalized to TSP) across samples.

**Figure 3:**
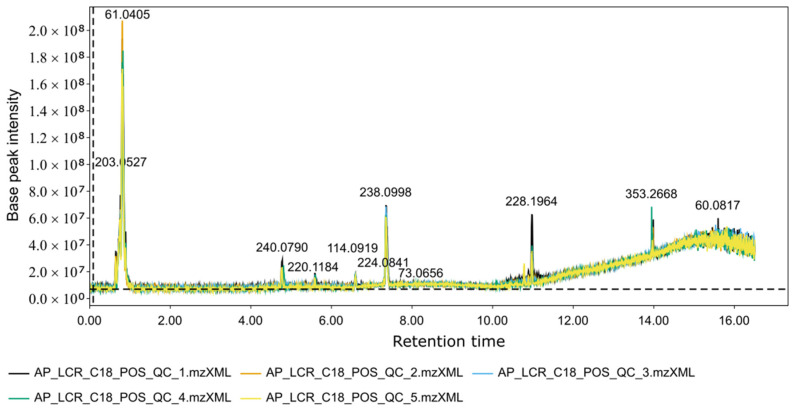
Total ion chromatogram (TIC) of QC samples acquired on the C18 ESI+ mode. Base-peak intensity is given in arbitrary units, and retention time is expressed in minutes.

**Figure 4:**
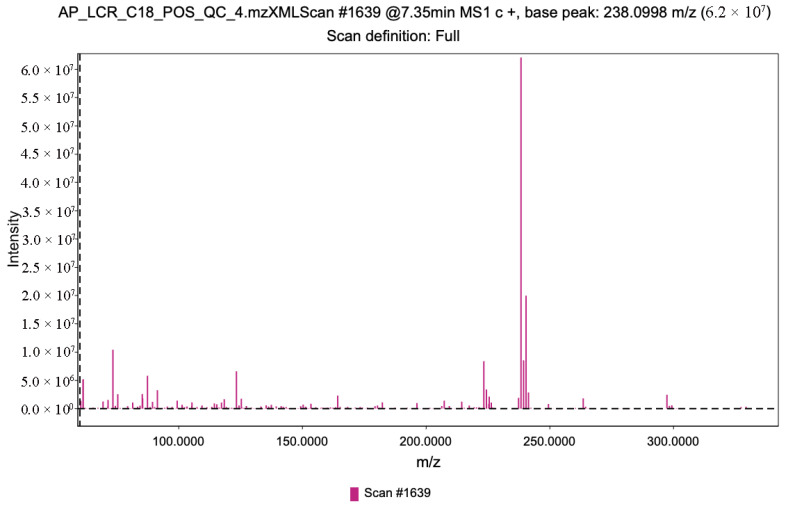
Example of a chromatographic scan at 7.35 min in a C18 ESI+ QC sample.

Each retention time corresponds to a series of scans capturing ion fragments related to a given metabolite (Figure 3). Each chromatographic peak is associated with a specific mass-to-charge (m/z) value (Figure 4). Together, retention time (RT) and m/z pairs constitute the fundamental identifiers used for metabolite annotation.

Table 1 provides an overview of the metabolites detected and quantified in the CSF prior to redundancy filtering (Table 1). Approximately 50% of the redundancy was removed using the CV threshold criterion.

In Table 2, creatine appears across all four analytical modalities, illustrating redundancy in the dataset. To address this, the CV calculated from QC samples is used to determine which modality provides the highest reproducibility and, therefore, the most reliable signal across platforms. HPLC-MS C18 ESI^+^ modality exhibited the lowest CV and was consequently retained in the final matrix. KEGG identifiers are then added to facilitate downstream biological interpretation.

**Figure 5:**
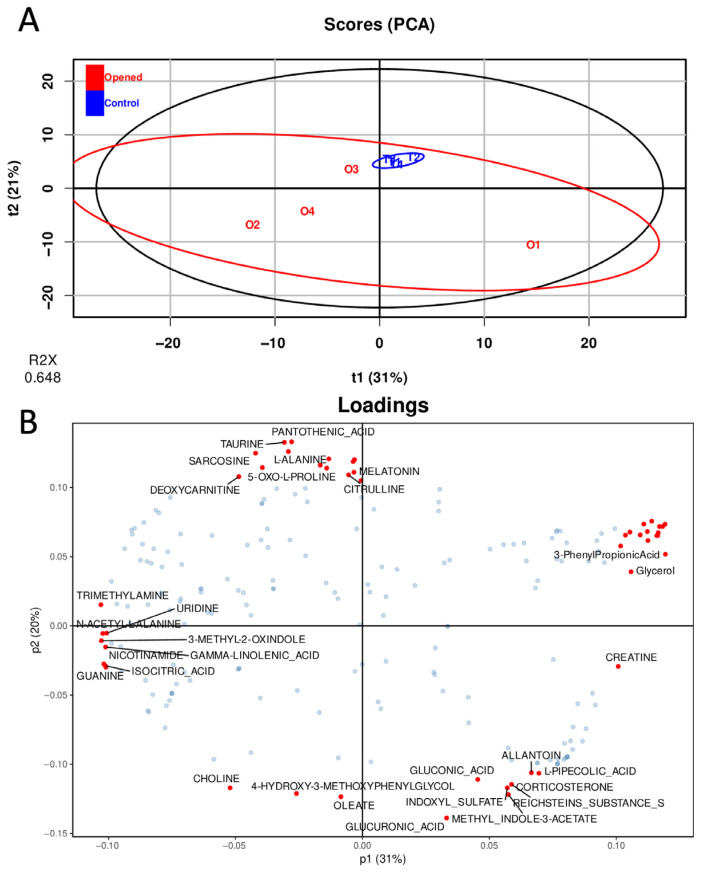
Unsupervised multivariate analysis of CSF samples. (**A**) Score plot and (**B**) loadings plot from a PCA performed on 209 quantified metabolites in eight CSF samples. In PCA, R2X denotes the explained variance of the dataset. It represents the proportion of total variance in the predictor matrix (e.g., metabolite concentrations) accounted by the principal components included in the model. Higher R^2^X values indicate that the PCA model captures a greater share of the dataset’s variability.

**Figure 6:**
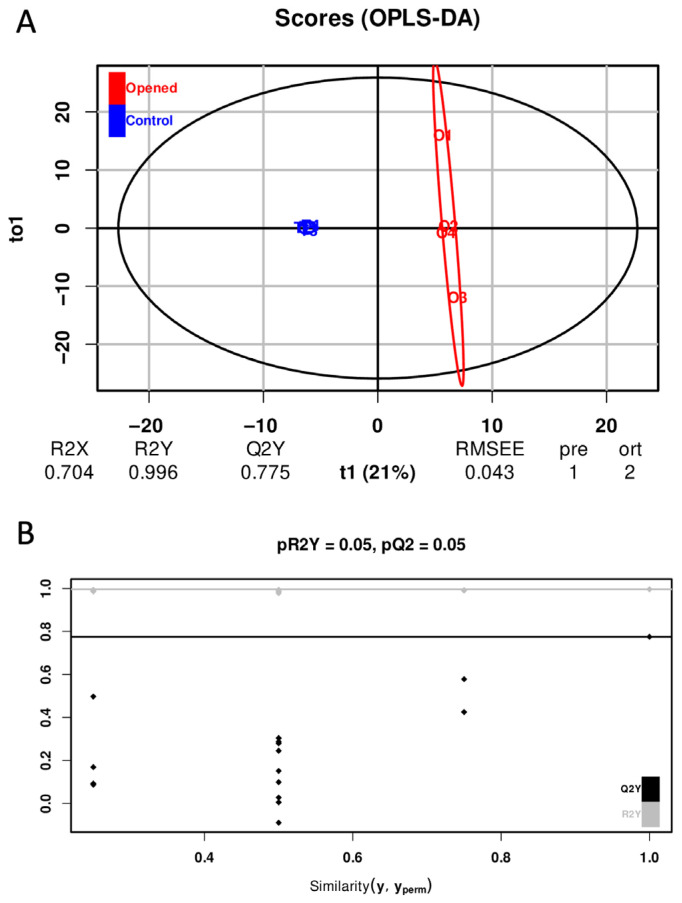
Supervised multivariate analysis. OPLS-DA (**A**) score plot and (**B**) permutation metrics based on 209 quantified metabolites in eight CSF samples. In OPLS-DA, several metrics are used to evaluate model performance and interpretability. R^2^Y measures the proportion of variance in the response matrix Y (e.g., class labels) explained by the model; higher values indicate stronger class separation. Q^2^Y represents the fraction of variance in Y that can be predicted by the model, typically assessed through cross-validation or permutation testing. Higher Q^2^Y values denote stronger predictive ability, with values above 0.5 generally considered acceptable. RMSEE (Root Mean Square Error of Estimation) quantifies the average deviation between observed and predicted Y values in the training set, with lower RMSEE values indicating a better fit. PRE (Predictive components) refers to the number of components in Y that contribute to prediction. ORT (Orthogonal components) corresponds to the components in X capturing variance unrelated to Y, thereby separating predictive from non-predictive variation and improving overall model interpretation.

Each individual sample is represented in the scores plot based on the first two principal components, with each point labeled according to its sample name (Figure 5A). The first and second components capture 31% and 21% of the total variance, respectively, explaining 52% of the variability in the dataset. The distribution of samples forms an ellipse reflecting the inter-individual heterogeneity within the overall metabolic pattern. PCA analysis reveals a clear and compact clustering of control animals, whereas rats subjected to transient BBBO exhibit a more dispersed distribution, indicating greater inter-individual variability in their metabolic response. The loadings plot (Figure 5B) illustrates the variables contributing to the principal components. While the scores plot displays the sample positions in the PCA space, the loading plot identifies the metabolites driving those positions. Variables located farther from the origin have stronger contributions to the components, and the angles between their vectors represent correlations (close alignment indicates a positive correlation, while the opposite orientation indicates a negative correlation). In this study, the loading plot provides a global view of the variables underlying the observed clustering. Further discrimination between groups can be obtained using supervised methods, such as OPLS-DA (Figure 6), which infer class membership based on experimental groups.

OPLS-DA is a supervised multivariate statistical method used to model differences between predefined groups. In contrast to PCA, which is unsupervised and captures only the directions of maximum variance within the dataset, OPLS-DA partitions the variation into two components: (i) predictive variation, which is correlated with group membership, and (ii) orthogonal variation, which is unrelated to group differences. This separation enhances model interpretability and facilitates the identification of variables (e.g., metabolites) that drive class discrimination. In an OPLS-DA score plot, groups appear clearly separated because the method explicitly optimizes discrimination, underscoring the need for rigorous model validation to avoid misinterpretation or overfitting. A permutation test is therefore required to verify that the observed separation reflects a true biological difference rather than chance. The procedure involves repeatedly permuting group labels (typically 100–1000 permutations); rebuilding the OPLS-DA model for each permutation; and recalculating performance metrics, such as R^2^ and Q^2^. The metrics of the original model are then compared with the distribution of permuted metrics using a statistical test. If the original model’s R^2^ and Q^2^ are significantly higher (with pR2Y and pQ2 representing *p*-values), the model is considered robust and unlikely to reflect random structure. In our study, the OPLS-DA model achieved a Q^2^Y value of 0.775, exceeding the commonly accepted threshold of 0.5 and indicating strong predictive performance (Figure 6A). The permutation test confirmed the validity of the model (pR^2^Y = 0.05; pQ^2^ = 0.05) (Figure 6B). Variable Importance in Projection (VIP) scores were used to quantify each metabolite’s contribution to the class separation; metabolites with a VIP > 1 were considered discriminant. Eighty-six metabolites met this criterion and were subsequently subjected to univariate analysis using the non-parametric Dunn test with Bonferroni correction. After statistical filtering, 26 metabolites were significantly different between the two experimental groups. Table 3 presents the top 10 discriminant metabolites with their log_2_ fold-change values and adjusted *p*-values (Table 3), while all significant metabolites are listed in Appendix A (Table A1).

Biological interpretation of metabolomics data relies on pathway analysis, which situates metabolites within metabolic networks to explain phenotypes. These analyses use curated databases, such as KEGG, HMDB, and Reactome [25,26,27]. Using the KEGG database, a hypergeometric test was performed as part of ORA (Table 4) [28]. This test evaluates the probability that a set of metabolites is statistically over-represented within a pathway. Raw *p*-values are adjusted using the False Discovery Rate (FDR) correction, with significance set at an adjusted *p*-value of <0.05.

Only the arginine biosynthesis pathway reached statistical significance after FDR correction (*p* = 4.44 × 10^−4^; FDR = 0.035). Alanine, aspartate, and glutamate metabolism (*p* = 3.60 × 10^−3^; FDR = 0.144) and tryptophan metabolism (*p* = 0.010; FDR = 0.284) exhibited the lowest raw *p*-values, suggesting potential enrichment trends (Table 4) as the FDR values did not reach significance. A complete table can be found in (Appendix A—Table A2). These preliminary findings indicate that arginine and related amino-acid pathways may be affected. Arginine plays a key role in reducing reactive oxygen and nitrogen species, supporting energy metabolism, regulating vascular tone, and modulating immune functions [29,30,31]. This versatile amino acid participates in numerous biological processes and can be converted into proline and glutamate [29]. Through its metabolic pathways, arginine contributes to protein synthesis, nitric oxide (NO) production, creatine synthesis, and polyamine biosynthesis—processes essential for cell growth, proliferation, and differentiation. It also supports nitrogen detoxification via the urea cycle. Because endothelial cells are the first targets of microbubble-assisted ultrasound [32], arginine is readily used by endothelial arginase, directing its metabolism towards the urea cycle, polyamine formation and the synthesis of proline or glutamate from ornithine. Arginine is also catabolized by nitric oxide synthase (NOS) to generate NO and citrulline, a reaction requiring cofactors, such as NADPH, FMN, FAD, and tetrahydrobiopterin. As the precursor of NO, arginine contributes to vasodilation, angiogenesis, and immune cytotoxicity [33]. Moreover, arginine availability influences macrophage polarization toward M1 or M2 phenotypes [31]. Analysis of CSF metabolism indicates that acoustically mediated BBBO disrupts arginine metabolism. These findings suggest that the procedure alters metabolic processes within the endothelial cells. Further investigation of arginine metabolism is therefore warranted, including mechanistic analyses of the pathways involved. Increasing the number of animals or developing approaches enabling repeated CSF sampling over time would help refine these conclusions. Our multiplatform analytical workflow is optimized for the discovery and quantification of polar metabolites in central metabolic pathways (e.g., amino-acid and carbohydrate metabolisms). However, protein precipitation with cold methanol does not efficiently extract apolar metabolites, such as lipids. Although lipids remain present in the sample, excessive lipid content can contaminate the NMR spectral signal and distort the baseline. CSF is naturally rich in phospholipids, glycerolipids, cerebrosides, ceramides, lysolipids, and related lipid classes [34,35,36]. Implementing a biphasic extraction protocol (e.g., Matyash MTBE, Bligh–Dyer, or Folch) would therefore be valuable to incorporate a lipidomic dimension into the analysis.

## 5. Reagent Setup

Phosphate Buffer Preparation: dissolve K_2_HPO_4_ and KH_2_PO_4_ in D_2_O to reach pH 7.4.

TSP Solution: prepare TSP solution in D_2_O at a final concentration of 3.2 mM.

## Figures and Tables

**Table 1:** Overview of the number of detected, identified, and quantified metabolites per analytical modality before and after redundancy filtering.

Modality	Number of Identified Metabolites	After Redundancy Filtering
C18 ESI^−^	78	45
C18 ESI^+^	109	40
HILIC ESI^+^	119	82
NMR	124	42
**Total**	**430**	**209**

**Table 2:** Example of redundancy filtering using CV values for creatine and L-tryptophan across analytical platforms.

Metabolites	QC1	QC2	QC3	CV	Modality	KEGG_ID
Creatine	0.001731	0.00124848	0.00152248	6.20102621	NMR	C00300
Creatine	298,417.412	335,617.359	316,098.658	17.0205854	C18_NEG	C00300
**Creatine**	**3.02 × 10^7^**	**4.09 × 10^7^**	**2.48 × 10^7^**	**3.91624533**	**C18_POS**	**C00300**
Creatine	1.57 × 10^8^	1.43 × 10^8^	1.34 × 10^8^	12.3868207	HILIC_POS	C00300
L-Tryptophan	445,183.068	499,114.178	514,065.011	13.4166623	C18_NEG	C00078
**L-Tryptophan**	**1.13 × 10^7^**	**7,859,753.25**	**9,668,132.3**	**5.57298324**	**C18_POS**	**C00078**
L-Tryptophan	5,429,491.27	5,450,753.5	5,993,301.75	17.6010797	HILIC_POS	C00078

Rows in bold are the modality retained according to the metabolites’ lower CV across the different modalities.

**Table 3:** Top ten metabolites ranked by VIP score from the validated OPLS-DA model.

Metabolites	VIP	KEGG ID	Log2 Fold Change	Adjusted *p*-Value
3-Hydroxyphenylacetate	1.957187	C05593	−1.2429992	0.0209
Galactitol	1.927569	C01697	−1.5931366	0.0209
L-Arginine	1.894037	C00062	1.9119724	0.0209
N-Methyltryptamine	1.883518	C06213	1.1507695	0.0209
3,4-Dihydroxy-l-phenylalanine	1.828094	C00355	−1.6887611	0.0209
L-Glutamine	1.772130	C00064	−1.8219156	0.0209
3-Methyladenine	1.718375	C00913	−1.3795471	0.0421
L-Asparagine	1.716927	C00152	−0.6577911	0.0209
Urate	1.715544	C00366	−1.7280361	0.0209
Lactate	1.696666	C00186	0.6132022	0.0209

**Table 4:** Metabolic pathways associated with the top ten metabolites.

Pathways	Total	Expected	Hits	Raw *p*	−log10(*p*)	FDR
**Arginine biosynthesis**	**14**	**0.16279**	**3**	**0.00044418**	**3.3524**	**0.035534**
Alanine, aspartate, and glutamate metabolism	28	0.32558	3	0.0036087	2.4426	0.14435
Tryptophan metabolism	41	0.47674	3	0.010678	1.9715	0.28475
Ascorbate and aldarate metabolism	10	0.11628	1	0.11069	0.95591	1
Arginine and proline metabolism	36	0.4186	1	0.34683	0.45988	1
Tyrosine metabolism	42	0.48837	1	0.39219	0.4065	1

Rows in bold are the pathways that are significantly dysregulated according to an FDR of <0.05.

## Data Availability

Data supporting the findings of this study are available from the corresponding authors on request.

## Abbreviations

The following abbreviations are used in this manuscript:

^1^H-NMRS	Nuclear magnetic resonance spectroscopy of proton
AD	Alzheimer’s disease
AU	Arbitrary unit
AUC	Area under the curve
BBB	Blood–brain barrier
BBBO	Blood–brain barrier opening
BL	Burst length
CNS	Central nervous system
CSF	Cerebrospinal fluid
CV	Coefficient of variation
D_2_O	Deuterium oxyde
ESI	Electrospray ionization
FAD	Flavin adenine dinucleotide
FC	Fold change
FMN	Flavin mononucleotide
FDR	False discovery rate
FID	Free induction decay
HILIC	Hydrophilic interaction liquid chromatography
HMDB	Human metabolome database
HPLC-MS	High-performance liquid chromatography coupled to mass spectrometry
KEGG	Kyoto encyclopedia of genes and genomes
LC-MS	Liquid chromatography coupled to mass spectrometry
LC-MS/MS	Liquid chromatography coupled to tandem mass spectrometry
MeOH	Methanol
MS	Mass spectrometry
m/z	Mass-to-charge ratio
NO	Nitric oxide
NOS	Nitric oxide synthase
NMR	Nuclear magnetic resonance
OPLS-DA	Orthogonal partial least square discriminant analysis
ORA	Over-representation analysis
PCNSL	Primary central nervous system lymphoma
PCA	Principal component analysis
PLS-DA	Partial least square discriminant analysis
PRF	Pulse repetition frequency
QC	Quality control
RBCs	Red blood cells
RMSEE	Root mean square error of estimation
ROCs	Receiver operating characteristic (ROC) curves
RT	Retention time
SNR	Signal-to-noise ratio
TIC	Total ion chromatogram
TOC	Total organic carbon
TSP	Trimethysilylpropionate
UPLC	Ultra performance liquid chromatography
VIP	Variable importance in projection

## Appendix A

**Table A1:** Significantly differential metabolites identified in CSF after univariate analysis (Kruskal–Wallis test with Bonferroni *p*-value adjustment).

Metabolites	Statistic	Adj *p*-Value	Modality	KEGG ID	VIP	Log_2_FC
3-Hydroxyphenylacetate	−2.309401	0.02092134	C18 NEG	C05593	1.957187	−1.2429992
Galactitol	2.309401	0.02092134	C18 POS	C01697	1.927569	1.1507695
L-Arginine	−2.309401	0.02092134	HILIC POS	C00062	1.894037	−1.6887611
N-Methyltryptamine	−2.309401	0.02092134	HILIC POS	C06213	1.883518	−1.7280361
3,4-Dihydroxy-L-Phenylalanine	2.309401	0.02092134	HILIC POS	C00355	1.828094	1.9119724
L-Glutamine	−2.309401	0.02092134	HILIC POS	C00064	1.772130	−1.3795471
3-Methyladenine	−2.032863	0.04206641	C18 POS	C00913	1.718375	−1.5931366
L-Asparagine	−2.309401	0.02092134	HILIC POS	C00152	1.716927	−1.8219156
Urate	2.309401	0.02092134	C18 POS	C00366	1.715544	0.6132022
Lactate	−2.309401	0.02092134	C18 NEG	C00256	1.696666	−0.6577911
Citrulline	−2.309401	0.02092134	HILIC POS	C00327	1.606109	−1.0026370
Indole-3-Acetic acid	−1.984313	0.04722090	HILIC POS	C00954	1.561969	−0.4700540
Citramalate	−2.309401	0.02092134	C18 NEG	C00815	1.550854	−0.7828884
Serotonin	2.323271	0.02016457	HILIC POS	C00780	1.503526	2.1690942
Glucuronic acid	2.309401	0.02092134	C18 NEG	C00191	1.499418	2.0298188
L-Alanine	−2.020726	0.04330814	HILIC POS	C00041	1.442019	−0.7555329
L-Lysine	2.309401	0.02092134	C18 POS	C00047	1.433950	0.1621405
Gluconic acid	2.020726	0.04330814	C18 NEG	C00257	1.421728	0.6382347
5-Oxo-L-Proline	−2.020726	0.04330814	HILIC POS	C01879	1.419276	−0.7024403
Indoxyl Sulfate	2.309401	0.02092134	C18 NEG	C05658	1.309764	2.6912877
5-Aminopentanoate	2.366432	0.01796048	C18 POS	C00431	1.130362	4.0047618
L-Norvaline	2.366432	0.01796048	C18 POS	C01826	1.130362	4.0047618
Methyl Indole-3-Acetate	2.020726	0.04330814	C18 POS	C20635	1.115888	3.4888185
Allantoin	2.309401	0.02092134	C18 NEG	C01551	1.091148	0.4922073
Corticosterone	2.020726	0.04330814	HILIC POS	C02140	1.085298	2.7098273
Reichstein’s Substance S	2.020726	0.04330814	HILIC POS	C05488	1.085298	2.7098273

**Table A2:** Pathway enrichment analysis of differential metabolites identified in CSF.

Pathways	Total	Expected	Hits	Raw *p*	−Log_10_(*p*)	FDR
Arginine biosynthesis	14	0.16279	3	0.00044418	3.3524	0.035534
Alanine, aspartate, and glutamate metabolism	28	0.32558	3	0.0036087	2.4426	0.14435
Tryptophan metabolism	41	0.47674	3	0.010678	1.9715	0.28475
Ascorbate and aldarate metabolism	10	0.11628	1	0.11069	0.95591	1
Arginine and proline metabolism	36	0.4186	1	0.34683	0.45988	1
Tyrosine metabolism	42	0.48837	1	0.39219	0.4065	1
Pentose and glucuronate interconversions	19	0.22093	1	0.20031	0.69829	1
Pyruvate metabolism	23	0.26744	1	0.23735	0.62462	1
Pentose phosphate pathway	23	0.26744	1	0.23735	0.62462	1
Steroid hormone biosynthesis	80	0.93023	2	0.23754	0.62426	1
Purine metabolism	71	0.82558	2	0.19837	0.70252	1
Glutathione metabolism	28	0.32558	1	0.28136	0.55074	1
Nitrogen metabolism	6	0.069767	1	0.067877	1.1683	1
Biotin metabolism	10	0.11628	1	0.11069	0.95591	1
Selenocompound metabolism	20	0.23256	1	0.20973	0.67835	1
Galactose metabolism	27	0.31395	1	0.27275	0.56423	1
Lysine degradation	30	0.34884	1	0.29829	0.52537	1
Inositol phosphate metabolism	30	0.34884	1	0.29829	0.52537	1
Glyoxylate and dicarboxylate metabolism	32	0.37209	1	0.31483	0.50192	1
Pyrimidine metabolism	39	0.45349	1	0.3699	0.43192	1
